# Predicting Rapid, Climate-Driven Shifts in North American Habitat Suitability for the Purple Pitcher Plant (*Sarracenia purpurea* L.)

**DOI:** 10.3390/plants14213337

**Published:** 2025-10-31

**Authors:** Christian H. Brown, Benjamin L. Frick, Jacqueline E. Mohan

**Affiliations:** Odum School of Ecology, University of Georgia, Athens, GA 30602, USAjmohan@uga.edu (J.E.M.)

**Keywords:** biogeography, climate change, conservation, global change ecology, habitat suitability, purple pitcher plant, *Sarracenia purpurea* L., wetland ecology

## Abstract

Climate change is shifting where suitable habitats occur for many species across the planet. *Sarracenia purpurea* L., the most widely distributed pitcher plant species in North America, already faces significant threats from land use change. While *S. purpurea* is well studied at physiological and local scales, threat assessments for this species at biogeographic scales are absent. Here, we remedy this by using Habitat Suitability Models to predict current suitable habitats and estimate climate-based shifts in the suitable habitat for *S. purpurea* in the near (2040) and long term (2100). The models predicted large areas of habitat loss in the southeastern United States and the western portion of the Great Lakes region by 2040. While the models also predict significant gains in suitable habitats north of the current *S. purpurea* range, the limited dispersal ability of this species precludes the possibility of natural migration to newly suitable habitats. Our results suggest that the degradation of considerable portions of current suitable habitats is already occurring and will continue in the future. Particularly threatened are the southern subspecies (e.g., *Sarracenia purpurea* subsp. *venosa*) of *S. purpurea*. We therefore urge land managers to make conservation efforts targeting threatened subspecies and encourage further the biogeographic investigation of less widely distributed congenerics of *S. purpurea*.

## 1. Introduction

Climate change continues to rapidly alter abiotic conditions on a global scale. Resulting modifications to rainfall, temperature, and seasonality have been shown to influence the suitability of native habitats for numerous taxa [1,2,3]. As a result, the shifting of species distributions in response to such environmental changes has become a necessary focus for the effective future conservation of many species [4,5,6]. Plants frequently play critical roles in defining and maintaining community structures, and consequently represent a salient point of focus for research aimed at understanding how changes in habitat suitability can initiate, facilitate, and/or inhibit distribution shifts on larger scales [2,7,8,9]. Intensified study in this area over the past several decades has revealed nuanced and largely inconsistent patterns of movement among many plant taxa in response to climate-driven shifts in habitat suitability [1,5,10,11]. Given this variability and the overall intricacy of plant dispersal at large scales, the identification of potential distribution shifts for specific plant taxa remains dependent on individualized habitat suitability assessments conducted in the context of their life histories and native ranges [4,11]. This is particularly true of specialist plant taxa, which generally rely on stricter environmental parameters and often inhabit rare or spatially isolated habitats. Due to these physiological and dispersal constraints, specialist plants are disproportionately affected by rapid abiotic shifts within their native ranges when compared with more generalist groups [12]. Despite this observation, relatively little attention has been paid to the potential dispersal responses of specialists in the context of climate change, and individualized habitat suitability assessments for these taxa remain invaluable from a conservation perspective [13,14].

Peatlands—freshwater wetlands characterized by anoxic, carbon-rich substrates—exist at specific intersections of temperature, precipitation, and nutrient availability [15,16]. Peatlands are common throughout North America but are frequently isolated, often being limited in extent by the depressional topography in which they form [17,18]. These habitats are further characterized by their resident plant communities—peatland plants are typically edaphic specialists, adapted to endure anoxic, inundated substrates, low nutrient availability, and in North America, temperate seasonality. Significant shifts in the abiotic regimes of North American peatlands have been observed over the past several decades, primarily defined by changes in temperature, precipitation, and the seasonality of both factors [19,20,21]. Alterations to the historical abiotic conditions of North American peatlands are especially pronounced in the southeastern United States, where more intense, but less frequent rainfall reduces groundwater recharge rates and moisture availability throughout the year [21,22]. Higher summer temperatures, elevated evapotranspiration, and a longer growing season exacerbate this trend, leading to wider variation in water table depth and more frequent periods of desiccation during summer months [15,18,22]. As peat substrates dry, decomposition accelerates, releasing previously limited nutrients at higher rates [15,23]. Increasing nutrient availability and drier substrates allow for other vascular plant species, often less-specialized forbs or even moisture-tolerant trees, to colonize historically unavailable habitat. This process further reduces water availability and limits the available habitat for peatland specialists [15,16]. Given their adaptations for the historical abiotic regimes of their habitats, the combined effects of altered precipitation seasonality, reduced water availability, and higher substrate decomposition rates in affected peatlands pose a threat to the long-term suitability of these habitats for specialist plants [24,25,26].

The purple pitcher plant (*Sarracenia purpurea* L.) is a widely distributed perennial herb native to North America and is commonly found in peatlands across the eastern United States and Canada (Figure S1). This species contains two recognized subspecies, the northern purple pitcher plant (*S. purpurea* subsp. *purpurea*) and the southern purple pitcher plant (*S. purpurea* subsp. *venosa*), each of which is further divided in several endemic varieties within their ranges [27]. The former subspecies is the only *Sarracenia* taxon to occur north of southeastern Virginia, USA (37° N) and the latter only occurs in the coastal plains of the southeastern United States [27]. There is currently significant debate as to whether these classifications are appropriate, but this study will consider *S. purpurea* subsp. *pupurea* and subsp. *venosa* as valid taxa. *S. purpurea* is well known for its distinct, pitcher-shaped leaves used to capture insect prey, a strategy which allows these plants to provide a reliable source of nutrients like nitrogen (N) and phosphorus (P) that are otherwise limiting in peatland systems. Like other peatland plants, *S. purpurea* is an edaphic specialist adapted to consistently inundated substrates, making it potentially vulnerable to desiccation during long periods of low water availability due to precipitation variability or invasion by other species. This is particularly true during lengthy maturation periods (~5 years), when immature *S. purpurea* are more vulnerable to environmental stochasticity [28,29]. Being a temperate plant, *S. purpurea* relies in part on temperature seasonality to properly time its phenology. Most pertinent is *S. purpurea’s* use of spring and winter temperatures as cues for germination, flowering, and initiating winter dormancy [30]. Earlier growing seasons in North American peatlands may impact the ability of *S. purpurea* to properly time both emergence and reproduction, potentially leading to ecological mismatches with pollinator species [15,20,31,32]. Previous research also indicates that elevated N concentrations in peatland substrates drastically reduce long-term population growth for *S. purpurea*, leading to higher extinction risks in affected populations [26]. Cumulatively, these findings suggest that climate-driven shifts in the abiotic regimes of peatlands—particularly in the southeastern United States—may alter the quality and extent of suitable habitats for *S. purpurea* in North America.

The life history and ecology of *S. purpurea* in North American peatlands suggests that this species is vulnerable to climate-based shifts in habitat suitability, namely those driven by changes in precipitation and temperature. A broad temperature tolerance but stringent precipitation requirements alongside a vast geographical range further makes *S. purpurea* well suited to large-scale modeling. Despite the proliferation of research on *S. purpurea* at local and organismal scales, the assessment of this species at biogeographical scales is lacking. Specifically, high-resolution maps of *S. purpurea* habitats and the associated drivers of habitat suitability are absent in the literature. The life history and dependence of *S. purpurea* on peatlands also suggests that the suitable habitat available to the group may undergo dramatic shifts due to climate change. Here we use Habitat Suitability Models (HSMs) to predict the extent of suitable habitat for *S. purpurea* in North America. Furthermore, we predict how this suitable habitat will be affected by climate change in the near term (2040) and long term (2100). Because of the rigid soil moisture requirements for *S. purpurea*, we hypothesize precipitation to be the primary driver of suitable habitat for the group. We expect that climate change-induced increases in temperatures will interact with more variable precipitation patterns to cause a net decrease in suitable habitats for *S. purpurea* both in the near and long term.

## 2. Results

### 2.1. Model Evaluation and Selection

Random forest models (RFs) performed best among the algorithms, followed by Gradient Boosted Models (GBMs), then Generalized Linear Models (GLMs) (Figure S2). Across all algorithms, all but 1 of the 27 individual models had True Skill Statistic (TSS) scores > 0.80 and area under the Receiver Operating Characteristic curve (ROC) scores > 0.90 and were therefore all included in ensemble averaging (Figure S2). One GLM had a TSS score < 0.80 and was therefore excluded from ensemble calculations (Figure S2). The committee average ensemble model was selected to represent current and future suitable habitat projections as it had the highest TSS and ROC scores relative to the mean and weighted mean ensemble models (Figure S3).

### 2.2. Environmental Variable Selection

Of the 30 tested environmental variables, 15 had variance inflation factors > 5 and were therefore eliminated from use in the Habitat Suitability Models (HSMs) (Table S1). Of the remaining 15 environmental variables, linear correlation coefficients varied from a minimal absolute value of 0.001–0.78. We therefore conclude that this set of environmental variables do not feature problematic collinearity issues [33] (Shrestha 2020). The most important variables were precipitation seasonality (i.e., the coefficient of variation in precipitation) and the precipitation of the warmest quarter of the year (i.e., in June, July, and August) (Table 1).

### 2.3. Habitat Suitability and Environmental Drivers

**Figure 1:**
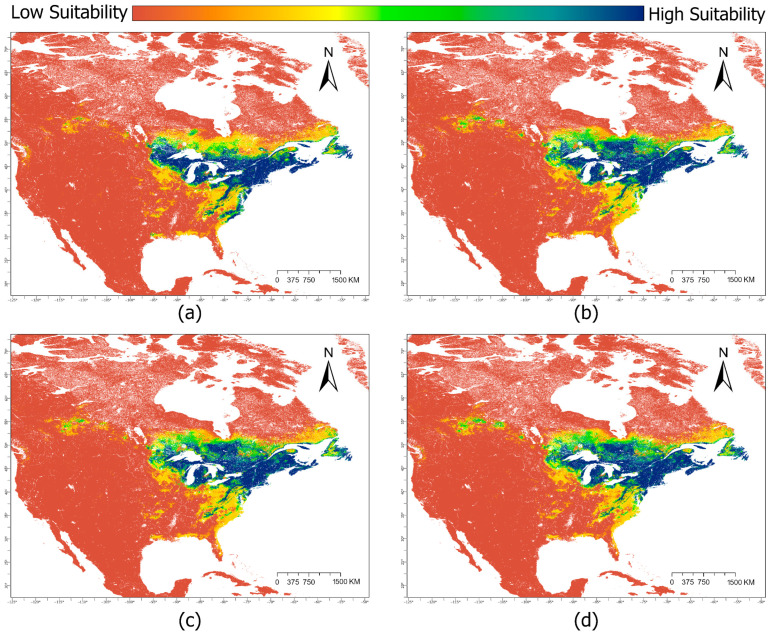
Suitable habitat of *Sarracenia purpurea* in North America for (**a**) the baseline climate scenario (climate normal period from 1970 to 2000) and three climate change scenarios projected to the year 2040: (**b**) Shared Socioeconomic Pathway (SSP) 1–2.6, (**c**) SSP 3–7.0, (**d**) SSP 5–8.5. Colors indicate habitat suitability.

**Figure 2:**
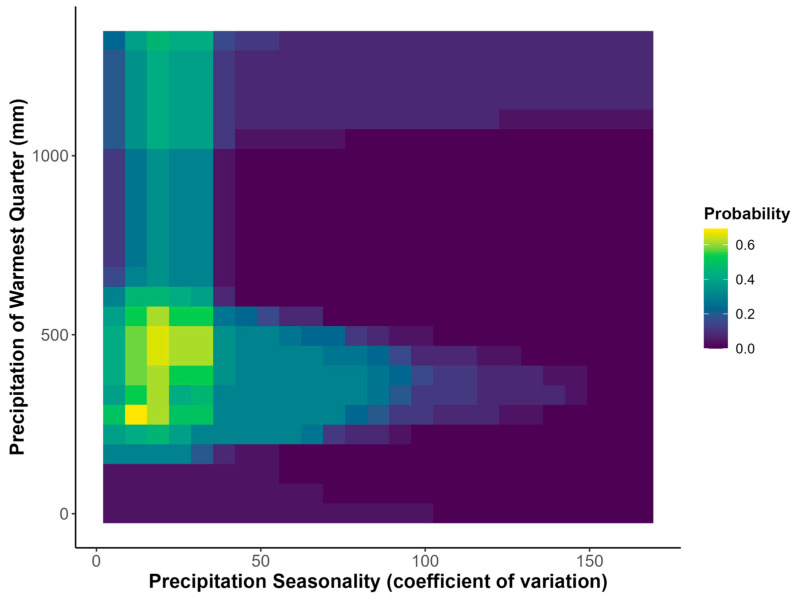
Bivariate response curve for precipitation seasonality (coefficient of variation) and precipitation of the warmest quarter of the year. Colors indicate the probability of suitable habitat occurring in response to each of the predictor variables.

The majority of suitable habitat for *Sarracenia purpurea* occurs around the Great Lakes region, as well as in the northeastern United States (Figure 1a). The range of suitable habitat extends northward to Nova Scotia and Newfoundland in Canada (Figure 1). Suitable habitat also extends, albeit in a more disjunct form, as far south as the southern Appalachian Mountains and the coastal plains of North and South Carolina in the United States (Figure 1). Response curves characterize the typical niche of *Sarracenia purpurea* and show that this species is relatively temperature insensitive and thrives in high year-round precipitation as well as acidic soils with a high soil carbon content (Figure S4). Bivariate response curves of precipitation seasonality and the precipitation of the warmest quarter show that the highest suitability of habitat occurs with low precipitation seasonality and between 250 and 500 mm of precipitation in the warmest quarter of the year (Figure 2).

### 2.4. Future Habitat Suitability

**Figure 3:**
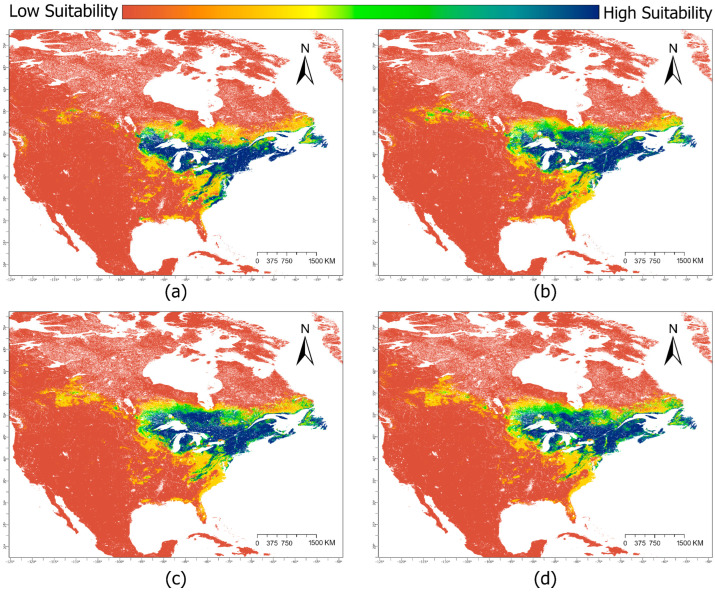
Suitable habitat of *Sarracenia purpurea* in North America for (**a**) the baseline climate scenario (climate normal period from 1970 to 2000) and three climate change scenarios projected to the year 2100: (**b**) Shared Socioeconomic Pathway (SSP) 1–2.6, (**c**) SSP 3–7.0, (**d**) SSP 5–8.5. Colors indicate habitat suitability.

Climate change scenarios projected to 2040 indicate a degradation in suitable habitats for the southern regions of *S. purpurea*’s distribution and a gain in new suitable habitat north and east of current leading-edge boundaries (Figure 1). These trends are enhanced in the 2100 projections with significant portions of the southern suitable habitat being entirely eliminated and further increases in suitable habitat to greater northeastern extents (Figure 3).

**Figure 4:**
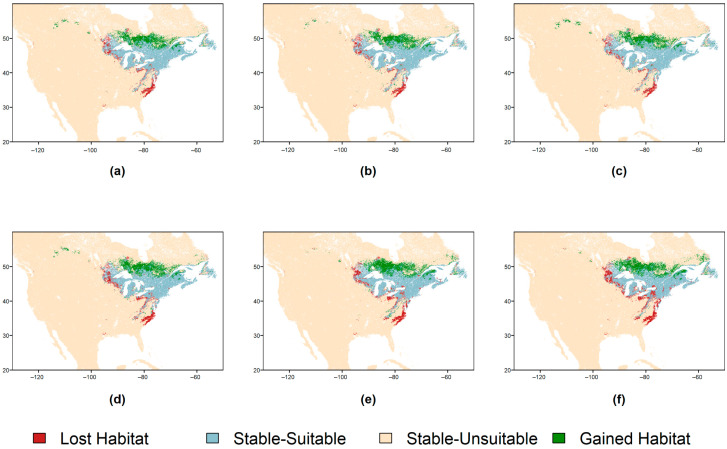
*Sarracenia purpurea* range change maps for six climate change scenarios: Shared Socioeconomic Pathway (SSP) 1–2.6, SSP 3–7.0, and SSP 5–8.5 by 2040 ((**a**–**c**), respectively) and 2100 ((**d**–**f**), respectively).

**Figure 5:**
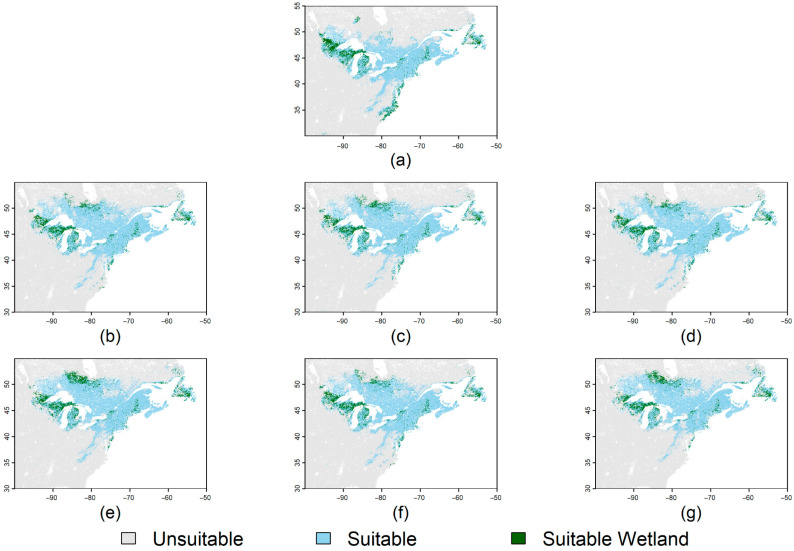
Intersection of binarized predicted suitable habitat with wetland land use classification. Suitable habitat is predicted across (**a**) the baseline climate, (**b**) Shared Socioeconomic Pathway (SSP) 1–2.6 by 2040, (**c**) Shared Socioeconomic Pathway (SSP) 1–2.6 by 2100, (**d**) SSP 3–7.0 by 2040, (**e**) SSP 3–7.0 by 2100, (**f**) SSP 5–8.5 by 2040, (**g**) SSP 5–8.5 by 2100. Suitable wetland habitat occurs where both predicted suitable habitat and wetland land use simultaneously co-occur.

Across all climate change scenarios, several overt trends are apparent. Most habitat loss occurs in the southern and western portions of the current suitable habitat range and most habitat gain occurs north of current habitat limits (Figure 4). Habitat loss ranged from 11 to 12% of total habitat area by 2040 and 14–21% loss by 2100 (Table 2). In the 2040 scenarios, Shared Socioeconomic Pathway (SSP) 1–2.6 (<3 °C warming by 2100) had the highest percentage of lost suitable habitat area, whereas in the 2100 scenarios SSP 5–8.5 (>5 °C warming by 2100) had the highest percentage suitable habitat area loss (Table 2). Habitat gain was greater than habitat loss during all climate normal periods and SSPs. The 2040 SSPs had gains in suitable habitat ranging from 25 to 26% of current suitable habitat area, with SSP 5–8.5 having the greatest increase (Table 2). In 2100, gains ranged from 28 to 31% with SSP 3–7.0 producing the greatest increase (Table 2). By subtracting lost area percentages from gained area percentages, the overall species range change is obtained which is a net gain of suitable habitat ranging from 9 to 15% increases across all SSPs (Table 2). It is important to note that the species range change values assume that *S. purpurea* can disperse to all new availhabitatsbitat. In the absence of dispersal, any new habitat essentially remains unsuitable and the suitable habitat loss terms would more accurately describe the overall change in suitable habitat. Intersections of suitable habitat across all climate scenarios with wetland areas show the vast majority of suitable wetland habitat occurs and increases northeast of the Great Lakes in Canada with climate change (Figure 5). Isolated patches of suitable wetland habitat also occur in the central Appalachian Mountains and the coastal Mid-Atlantic region in the United States in all scenarios, except SSP 5–8.5 (Figure 5).

## 3. Discussion

Our results present a clear, yet complex perspective on the future of *S. purpurea* in North American peatlands. Our initial hypothesis that precipitation would be the primary driver of habitat suitability shifts for *S. purpurea* was supported, which corroborates the idea that *S. purpurea* is sensitive to changes in water availability (Table 1). More specifically, our results point to the seasonality of precipitation and the mean precipitation of the warmest quarter being important climatic factors for determining the suitability of *S. purpurea* habitat (Figure 2). This finding supports the implication that alterations to historical precipitation seasonality in peatlands may be threatening to *S. purpurea*, and further that the species may be especially vulnerable to desiccation events during summer months.

Interestingly, the severity of each SSP scenario seemed to have relatively little impact on the overall trends observed between them (Table 2), and the same general pattern of habitat expansion and contraction was present in all projections (Figure 1, Figure 3 and Figure 4). Under the assumption that *S. purpurea*’s dispersal capability is adequate to colonize new habitat quickly, our expectation of a net loss in suitable habitat was unsupported. In this scenario, all climate projections in this study depict a net increase in suitable habitat for *S. purpurea.* Specifically, our projections highlight the retention of high-suitability habitat in the Great Lakes region and a northward expansion of suitable habitat from this core range. This assumption, however, seems unlikely to be met by *S. purpurea*’s notoriously low dispersal distance (~5 cm per reproductive event), which only occurs locally through seed floatation [29]. Additionally, despite the ubiquity of *S. purpurea* in isolated peatlands throughout many regions of North America, no long-distance dispersal mechanisms have ever been empirically identified for the species [29]. Previous research has suggested that wetland birds may play a role in dispersing *Sarracenia* seeds, but this potential mechanism remains unsupported [29]. Without a confirmed means to disperse over large distances, at present, it seems infeasible for the dispersal of *S. purpurea* spp. to match the rapid pace of the habitat expansions and contractions depicted in our projections. Considering this implication, identifying potential dispersal mechanisms and ascertaining long-distance dispersal rates in *S. purpurea* are paramount to accurately predicting how its overall range may shift in response to changes in habitat suitability.

The importance of climate variables for the conservation of rare and declining plant species is highlighted in previous work on the largest and most diverse family of flowering plants, the Orchidaceae [34]. Wang et al. [35] determined that mean annual precipitation, mean elevation, mean annual minimum temperature, and mean annual maximum temperature accounted for up to 97% of geo-referenced cells in central Texas for the presence/absence of an endemic orchid. Understanding the future risk to suitable peatland habitats will allow researchers and conservationists to identify areas most at risk and take appropriate management actions to ensure persistence.

Interpretations of our results are further complicated by the existence of latitudinally distinct subspecies and numerous endemic varieties within *S. purpurea*. *S. purpurea* subsp. *purpurea* and its associated varieties have a much wider range than *S. purpurea* subsp. *venosa*. Despite losses in high-suitability habitat at the western edge of the range, high frequencies of occurrence within core habitat maintained in the Great Lakes region suggest that observed shifts in habitat suitability may not pose as serious a threat to *S. purpurea* subsp. *purpurea* as they do to its sister taxon. Substantial losses in high-suitability habitat throughout the southeastern and midwestern United States (Figure 1, Figure 3 and Figure 4) imply both potential negative impacts for *S. purpurea* subsp. *venosa* populations within those regions and the future degradation of peatlands therein as habitat for specialist plants. The range of *S. purpurea* subsp. *venosa* is entirely contained within these projected losses, and so this subspecies and its constituent varieties may be disproportionally affected by climatic shifts. One potential exception to this trend is the endemic Appalachian variant of *S. purpurea* subsp. *venosa*, the mountain sweet pitcher plant (*S. purpurea* subsp. *venosa* var. *montana*). The range of *S. purpurea* subsp. *venosa* var. *montana* is also within degrading areas, but its core habitat is maintained through all projections (Figure 1 and Figure 3).

Our results may also imply rapidly shifting suitable habitat for *S. purpurea* congenerics that feature much narrower ranges of thermal or precipitation tolerance. The genus *Sarracenia* contains an additional 10 species, the ranges of which are all restricted to the southeastern United States. Several of these species (*S. leucophylla*, *S. alata*, *S. flava*, *S. jonseii*, *S. minor*, and *S. oreophila*) are frequently found in peatlands inhabited by *S. purpurea* and may also be subject to habitat suitability shifts within our study’s timeframe. Two of these species, *S. jonseii* and *S. oreophila*, are federally endangered within their native ranges, and as such are of particular concern in the context of shifting habitat suitability. While the exact nature of potential shifts in habitat suitability for these species would be dependent on their individual life histories, dispersal abilities, and current distributions, their more restrictive climatic niches may impose substantial losses in southeastern US habitat as climate change progresses. Individualized, large-scale habitat suitability assessments like those conducted in this study would be similarly beneficial in understanding how climate change may impact future conservation decisions for these species.

Given the complexity of plant–climate interactions at large scales, it is important to consider that residing in less suitable habitat does not necessarily imply immediate extirpation. *S. purpurea* populations in less suitable habitats may persist for some time despite their unfavorable circumstances, and directed conservation may be necessary to preserve or relocate them. Our results suggest that *S. purpurea* subsp. *venosa* and its endemic variants would likely benefit from intensified management and study, potentially also in the context of sea level rise and climate-driven storm events, which our projections did not consider. The majority of extant *S. purpurea* subsp. *venosa* habitats is in low-lying, coastal peatlands that are potentially vulnerable to saltwater intrusion and storm surges. Abbott and Battaglia [36] examined the effects of storm surge on one such peatland inhabited by *Sarracenia rosea* (formerly *S. purpurea* subsp. *burkii*), finding that saltwater exposure drastically reduced *S. rosea* cover and ultimately concluding that significant saltwater intrusion may severely inhibit the recovery of affected coastal peatland plant communities. All projections in this study suggest that, by 2040, the majority of remaining high-suitability habitat for *S. purpurea* subsp. *venosa* will be located along the Atlantic coast (Figure 1 and Figure 3), potentially leading to overlap with projected sea level rise and storm surge zones in low-lying peatlands [37]. Should this be the case, regions of otherwise highly suitable peatland habitat may be rendered intolerable for *S. purpurea* subsp. *venosa* by gradual saltwater intrusion or increasingly frequent storm surges. Additionally, this study did not consider projected habitat loss due to changes in land use by 2040 or 2100. Habitat loss due to anthropogenic activity represents the single greatest threat to *Sarracenia* species in North America, particularly in the United States [38]. From 1700 to 2020, ~50% of US wetland area has been lost, primarily through conversion to cropland and urban areas [18]. Assuming a continuation of historical trends, these land use changes will likely compound with those of climate change to further reduce the availability of high-suitability *Sarracenia* habitat.

The future of *Sarracenia* conservation in North America will be necessarily intensive and will further rely on the consideration of additional trends such as sea level rise and land use over long timescales. It is our recommendation that future *Sarracenia* conservation strategies emphasize the in situ preservation of naturally occurring and otherwise endemic *Sarracenia* varieties. Established ex situ and commercial conservation should be utilized to support the assisted migration and consolidation of populations through live introductions and seeding, but should not be relied upon entirely due in part to the risks associated with introducing clonal and greenhouse-reared plants to endemic populations [39,40,41]. Additionally, given the potential differences in climate optima between *S. purpurea* subspecies and any other co-occurring *Sarracenia* species, any recommendations for priority areas would be contingent on the original location of an affected population. Generally, however, sites should be based in large, unaltered, and contiguous peatlands situated away from both the Atlantic coast and any regional metropolitan areas. Larger peatlands are more resistant to variability in rainfall, so their prioritization may be a more efficient strategy for the long-term preservation of local genetic diversity than the management of many individual microsites. Microsite populations are more vulnerable to climate change, and will likely be the first to disappear as habitat suitability shifts progress [15,18,42]. Habitat managers along the southeastern coastal plain should emphasize the preservation of genetic diversity in local populations and seek to consolidate adjacent microsite populations into larger, more ecologically stable, and contiguous habitats. Determined seed and live rhizome collection efforts ahead of schedule will allow for an adaptive response as habitat suitability in microsites diminishes. Figure 5 depicts the current extent of wetland area in the mid- and north Atlantic regions of North America alongside predicted high-suitability *S. purpurea* for each SSP scenario tested in this study. Of the areas currently classified as extant freshwater wetlands, only those to the northeast of the Great Lakes region intersect with all projections of high-suitability habitat extent by 2100. Wetlands located on the southern edge of the Great Lakes region are maintained by 2100 in all projections except SSP 5–8.5, suggesting that potential sites for the conservation of *S. purpurea* subsp. *venosa* and other southern *Sarracenia* species should be strategically established further north of climatic optima to account for variability in projections. *Sarracenia* conservation sites should ideally encompass habitat with no history of human alteration—remote peatlands with no obvious economic value may provide some protection from future human interference. The locations of these sites, particularly those in more publicly accessible areas, should be closely guarded as poaching represents a serious threat to the efficacy of long-term conservation for *Sarracenia*. Stable populations rely on large, mature ramets to maintain recruitment, and these older plants are often the most attractive to would-be thieves [41].

This study highlights the continued need for research investigating the impacts of climate change on the distribution of suitable habitats for plant species and additionally demonstrates the value of large-scale biogeographical analyses for the conservation of specialist plant groups. Climate change interactions are complex—general trends do not always apply to individual species, particularly to those which inhabit restrictive environmental niches. Species-level habitat suitability assessments are extremely valuable in the conservation context for their ability to inform adequate and prompt conservation action. As climate change progresses in North America, such information might allow for the continued preservation of unique, charismatic, and ecologically significant flora.

## 4. Materials and Methods

Habitat Suitability Models (HSMs), also referred to as ecological niche models or species distribution models, relate the presence and absence of a focal species as a response to environmental predictors to project where suitable habitat might occur now or in the future [43]. Here, we use the term HSM as our goal is to model the current and future potential suitable habitat of *Sarracenia purpurea*, as opposed to its actual distribution. By altering the environmental predictors to expected future conditions, projections can be made regarding how a species’ suitable habitat might be affected by climate change [44]. The R package ‘biomod2’ (version 4.2-5-2) was used to enable an ensemble modeling approach for predicting current and future habitat suitable for *S. purpurea* [45]. Ensemble modeling, averaging outcomes from several models into one consensus model, was used to reduce the bias in predictions that may arise from selecting one or another algorithm [46]. This ensemble modeling approach is particularly useful in the cases where many algorithms produce well-fit models that would have to be arbitrarily chosen between to represent the data [43].

A total of 9786 *S*. *purpurea* presence points were obtained from the Global Biodiversity Information Facility (GBIF [47]) to be used as a response variable in HSMs [48] (Figure S1). Presence points only included those identified as human observations and iNaturalist research-grade observations in the GBIF. Presence points outside of *S. purpurea’s* native habitat of North America were excluded. Some presence points belonged to *Sarracenia rosea*, a former subspecies of *S. purpurea* (subsp. *venosa* var. *burkii*), which were likewise excluded from the models. To minimize spatial bias from citizen science data, presence points were thinned by eliminating points closer than the mean distance to the nearest neighboring point (3.20 km^2^), yielding a total of 4977 presence points [49]. Three random generations of 10,000 pseudo-absence points were used to inform models of the full environmental gradients. Multiple random generations of a large number of pseudo-absence points enabled any bias in pseudo-absence selection to be identified in model evaluations [50].

A total of 30 environmental predictors, including climate and soil variables, were vetted for inclusion in the HSMs (Table S1). Baseline (climate normal period: 1970–2000) and future climate variables were obtained from WorldClim.org [51]. Three climate change scenarios, Shared Socioeconomic Pathway (SSP) 1–2.6, SSP 3–4.0, and SSP 5–8.5, were applied in this study for predicted climate normals of 2021–2040 and 2081–2100. In terms of warming, SSP 1–2.6 represents a best-case scenario where warming is kept <3 °C by 2100, SSP 3–4.0 is a middle-of-the-road scenario with warming <4 °C, and SSP 5–8.5 is a worst-case scenario with warming exceeding 5 °C. In all climate change scenarios, we used NASA’s Goddard Institute of Space E2.1 climate model [10]. Soil variables were obtained from the International Soil Reference and Information Centre SoilGrids 2.0 dataset [52]. Soil variable values were obtained from a depth 15–30 cm, the typical rooting zone of *S. purpurea* (Pers. Obs.), and were kept constant across all climate scenarios. All environmental rasters had a resolution of 30 arc seconds (about 1 km^2^). We also intersected the predicted suitable habitat from our models with land area designated as ‘wetland’ from the Commission for Environmental Cooperation’s 30 m resolution (resampled to match the 30 arc second resolution of the remaining environmental data) land use map [53] (CEC 2024). This intersection map is meant to further qualify the areas where conservation efforts might most effectively be applied.

All rasters were clipped to the extent of North America. The total surface area modeled was over 26,018,150 km^2^, accounting for North America and some small extraneous areas kept due to clipping geometry. To best visualize our results, the extents displayed in our maps are a subset of this total area but still capture the full geographic distribution of suitable habitat for *S. purpurea* (Figure 1, Figure 3 and Figure 4). All presented area calculations were made on North American Albers Conical Equal Area projections of our maps.

To prevent issues of multicollinearity among environmental variables, a variance inflation factor (VIF) analysis test was applied. Any variables with a VIF score above 5 were considered too collinear and were excluded from the models [54]. Variable importance was calculated by randomly shuffling values of an environmental variable and measuring the resulting decrease in model prediction accuracy [55]. Response curves for environmental variables were created using the evaluation strip method [56].

Here, we used three algorithms—Generalized Linear Models (GLMs), random forests (RFs), and boosted regression trees, referred to as Gradient Boosted Machines/Models (GBMs) in ‘biomod2’—to inform our final ensemble models. All GLMs were run with polynomial terms. Random forests were run with 500 trees and five candidate environmental variables sampled at each branch. Finally, GBMs were run with 7500 trees, an interaction depth of 9, and a shrinkage value of 0.05. All other aspects of algorithm parameterization were left to their default setting in ‘biomod2’ (version 4.2-5).

Three cross-validation datasets were produced by randomly partitioning data into 80% calibration data and 20% evaluation data three times. For each algorithm a model was run for every unique combination of cross-validation dataset and pseudo-absence selection resulting in 27 total individual models (3 algorithms × 3 cross-validation datasets × 3 pseudo-absence selections).

Individual models were evaluated using True Skill Statistics (TSSs) and the area under the Receiver Operating Characteristic curve (ROC), both of which range in value from zero to one on the following scales. For TSS: poor 0.00–0.20; fair 0.21–0.40; moderate 0.41–0.60; substantial 0.61–0.80; and excellent 0.81–1.00 [49]. The TSS combines information from sensitivity, the errors of omission, and specificity, the errors of commission [57]. For ROC: counter-predictions < 0.50; fail 0.51–0.60; poor 0.61–0.70; fair 0.71–0.80; good 0.81–0.90; and excellent 0.91–1.00 [58]. All models that did not meet a combined threshold of TSS > 0.80 and ROC > 0.90 were excluded from ensemble model calculations.

Three types of model averaging were used in this study to create ensemble models: mean, weighted mean, and committee average. The mean ensemble model averaged raw prediction values of habitat suitability for a particular cell across individual models (GLMs, RFs, and GBMs that passed the TSS and ROC thresholds). The weighted mean ensemble model similarly averaged predictions across individual models; however a weight was assigned to each prediction based on its TSS score where a high TSS creates a corresponding high weight. The committee average ensemble model was generated by first transforming individual model predictions into a binary format using a cutoff value of prediction probabilities which maximized TSS scores, then averaging those binarized predictions.

Ensemble models were likewise evaluated on the TSS and ROC using the same scale as before. Predictions of all models are interpreted as the degree of habitat suitability as opposed to the actual distribution of *S. purpurea*, as randomly generated pseudo-absence points were used [59].

## Figures and Tables

**Table 1:** Environmental variable code, descriptions, and associated importance values and standard deviations.

Variable Code	Variable Description	Mean Importance	Standard Deviation
PrecipWarmQ	Precipitation of the Warmest Quarter	0.26	<0.01
PrecipSeasonality	Precipitation Seasonality (coefficient of variation)	0.09	<0.01
MeanTempDryQ	Mean Temperature of the Driest Quarter (°C)	0.09	<0.01
MeanDiurnalRange	Mean Diurnal Range (°C)	0.07	<0.01
bdod_1530_mean	Bulk Density of the Fine Earth Fraction (cg/cm^3^)	0.06	<0.01
phh2o_1530_mean	Soil pH (pH × 10)	0.03	<0.01
PrecipColdQ	Precipitation of the Coldest Quarter (mm)	0.03	< 0.01
clay_1530_mean	Proportion of Clay Particles (<0.0002 mm) in the Fine Earth Fraction (g/kg)	0.02	<0.01
cec_1530_mean	Cation Exchange Capacity of the Soil (mmol(c)/kg)	0.02	<0.01
cfvo_1530_mean	Volumetric fraction of coarse fragments (cm^3^/dm^3^, volume %)	0.02	<0.01
MeanTempWetQ	Mean Temperature of the Wettest Quarter (°C)	0.02	<0.01
ocs_1530_mean	Organic Carbon Stock (t/ha)	0.02	<0.01
silt_1530_mean	Proportion of Silt Particles (≥0.002 mm and ≤0.05/0.063 mm)	0.01	<0.01
N_1530_mean	Total Nitrogen (N) (cg/kg)	0.01	<0.01
soc_1530_mean	Soil Organic Carbon Content in the Fine Earth Fraction (dg/kg)	0.003	<0.01

**Table 2:** Suitable habitat losses, gains, overall range change, and total area of suitable habitat displayed compared to the baseline climate scenario. Range change is the difference between gain and loss values and assumes *Sarracenia purpurea* is able to disperse to all newly suitable habitat.

Scenario	Loss	Gain	Range Change	Total Area (Million km^2^)
Baseline Climate	-	-	-	14.09
SSP 1–2.6 2040	12.90%	26.19%	13.29%	15.39
SSP 1–2.6 2100	14.44%	28.24%	13.80%	15.37
SSP 3–7.0 2040	12.47%	25.07%	12.61%	15.33
SSP 3–7.0 2100	16.51%	31.56%	15.05%	15.54
SSP 5–8.5 2040	11.79%	25.93%	14.14%	15.52
SSP 5–8.5 2100	21.17%	31.08%	9.91%	14.58

## Data Availability

The raw data supporting the conclusions of this article will be made available by the authors on request.

## Supplementary Materials

The following supporting information can be downloaded at: https://www.mdpi.com/article/10.3390/plants14213337/s1, Figure S1: Presence points (n = 4977) of *Sarracenia purpurea* in North America. Presence points tagged as iNaturalist Research Grade Observations (i.e. community verified data) were obtained from GBIF.org (Telenius 2011) and used as a response variable in habitat suitability models; Figure S2: True Skill Statistic (TSS) and Area Under the Receiver Operator Characteristic Curve (ROC) evaluations for individual models, symbolized by algorithm. Algorithms include Gradient Boosted Models (GBMs), Generalized Linear Models (GLMs), and RandomForests (RFs). Clustered points were jittered for better visibility; Figure S3: True Skill Statistic (TSS) and Area Under the Receiver Operator Characteristic Curve (ROC) evaluations for ensemble models, symbolized by algorithm and colored by evaluation metric filter. Algorithms include Gradient Boosted Models (GBMs), Generalized Linear Models (GLMs), and RandomForests (RFs). Clustered points were jittered for better visibility; Figure S4: Response curves of *Sarracenia purpurea* habitat suitability to changes in environmental variables in the committee average ensemble model for environmental variables. Focal variables were held at their median values when implementing the evaluation strip method. Refer to Table S1 for variable descriptions and x-axis units; Table S1: Environmental variable codes, their descriptions and units, and Variance Inflation Factor (VIF) analysis scores.

## Abbreviations

The following abbreviations are used in this manuscript:

HSM	Habitat Suitability Model
GBIF	Global Biodiversity Information Facility
SSP	Shared Socioeconomic Pathway
VIF	Variance Inflation Factor
GLM	Generalized Linear Model
RF	Random Forest
GBM	Gradient Boosted Machine/Generalized Boosted Model
TSS	True Skill Statistic
ROC	Area under the Receiver Operating Characteristic curve
