# Supplementary material for: Predicting Rapid, Climate-Driven Shifts in North American Habitat Suitability for the Purple Pitcher Plant (Sarracenia purpurea L.)
Source: Plants (Basel). 2025 Oct 31;14(21):3337. doi: 10.3390/plants14213337 (PMC12608460; doi:10.3390/plants14213337)
Supplement: Supplementary file 1 [file plants-14-03337-s001.zip › plants-3874578-supplementary.pdf]

Supplemental Figures and Tables

SUPPLEMENTAL FIGURES

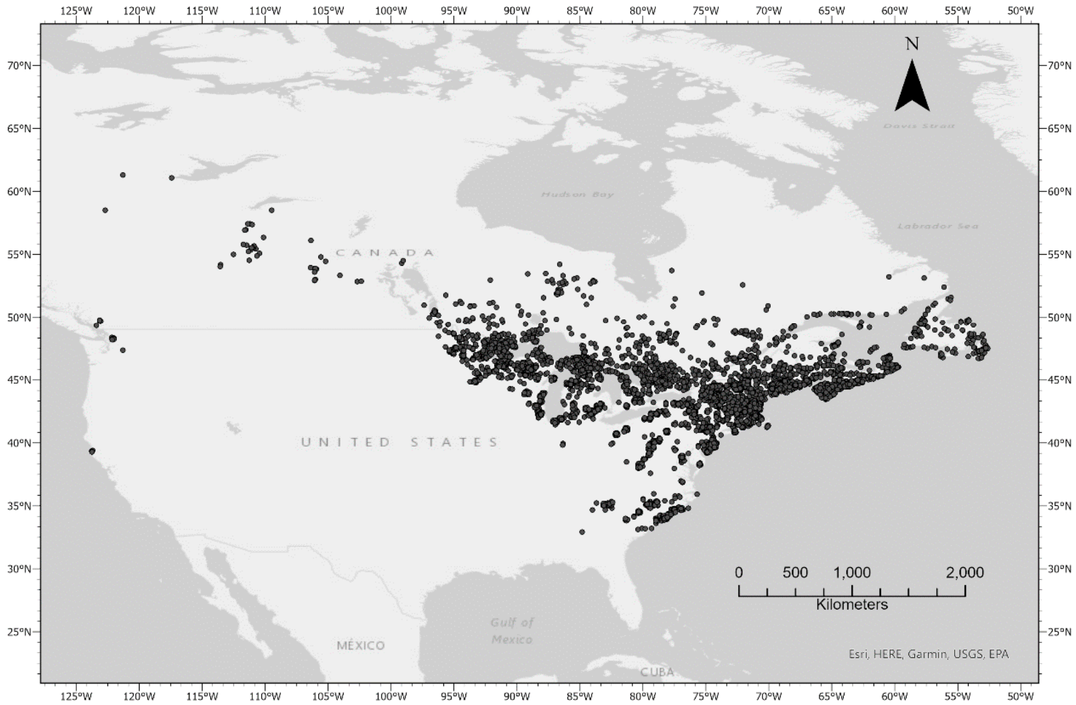

Figure S1 Presence points (n = 4,977) of *Sarracenia purpurea* in North America. Presence points tagged as iNaturalist Research Grade Observations (i.e. community verified data) were obtained from GBIF.org (Telenius 2011) and used as a response variable in habitat suitability models.

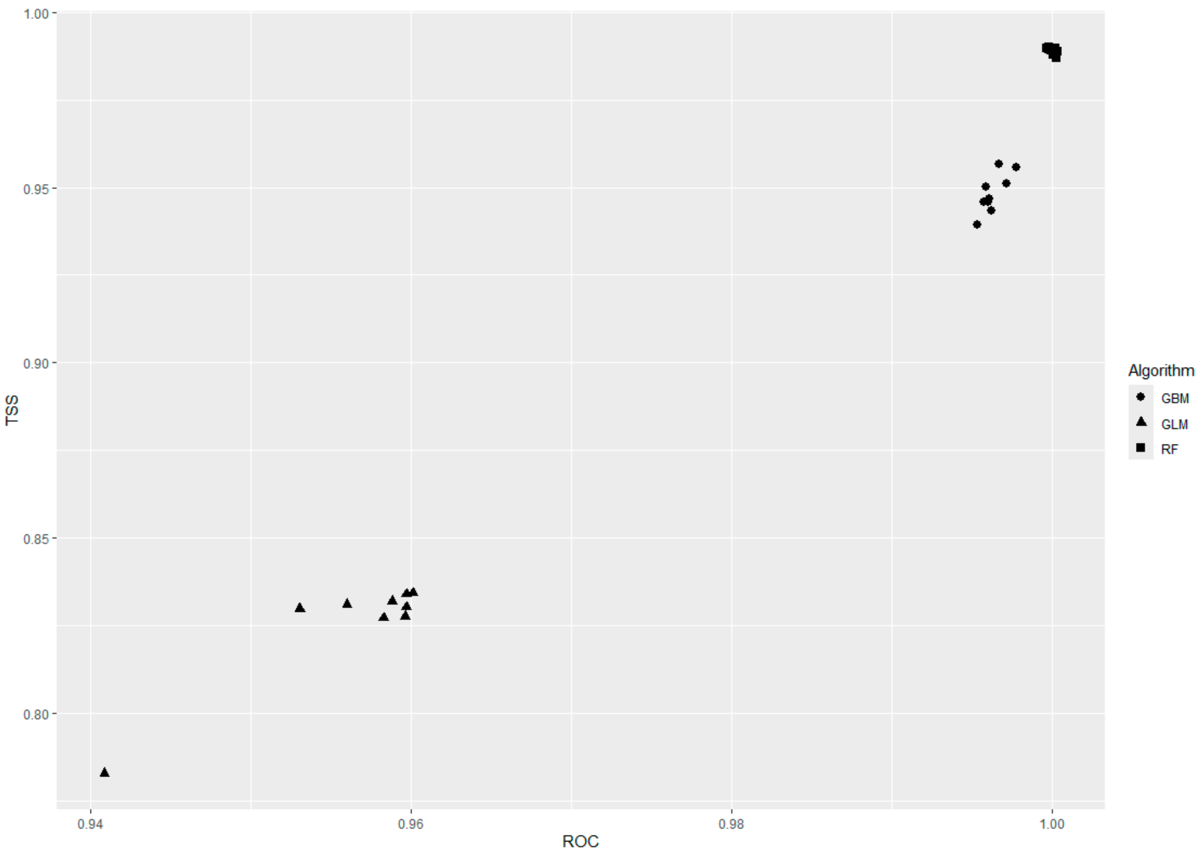

Figure S2 True Skill Statistic (TSS) and Area Under the Receiver Operator Characteristic Curve (ROC) evaluations for individual models, symbolized by algorithm. Algorithms include Gradient Boosted Models (GBMs), Generalized Linear Models (GLMs), and RandomForests (RFs). Clustered points were jittered for better visibility.

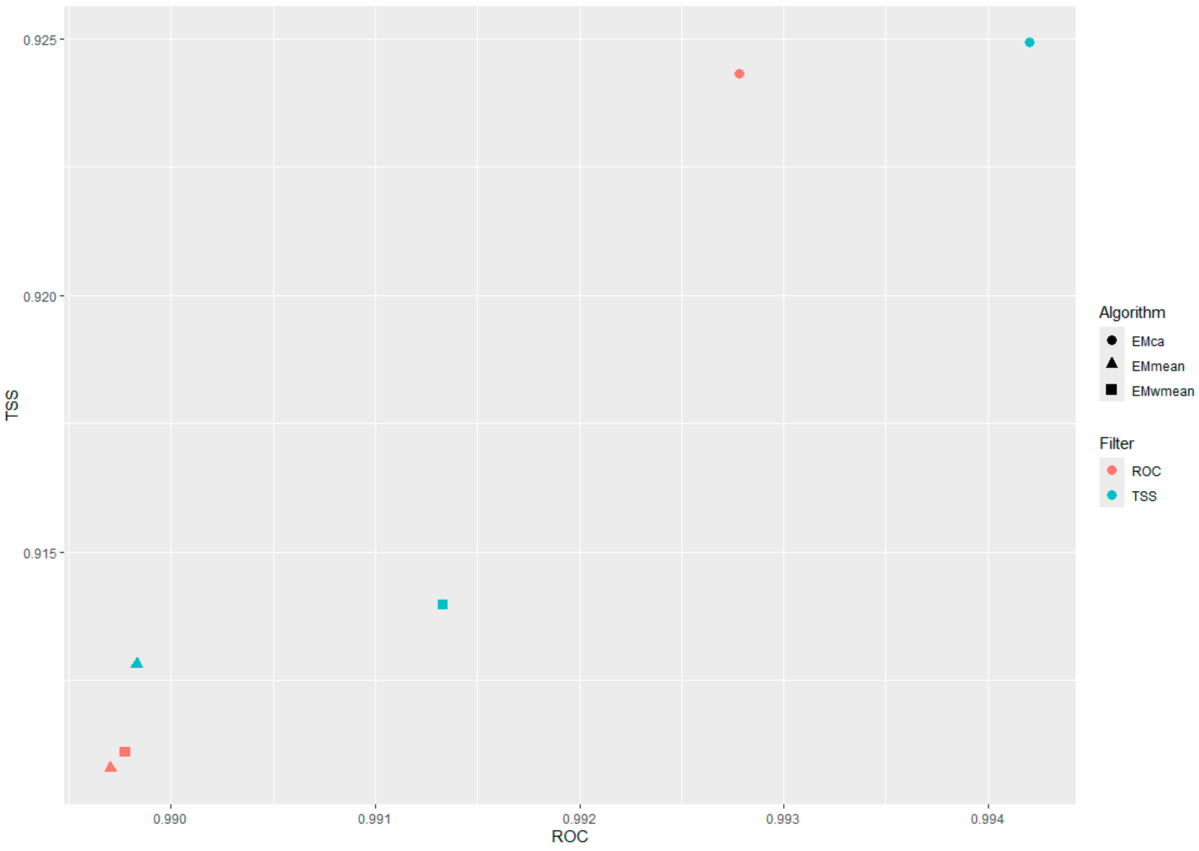

Figure S3 True Skill Statistic (TSS) and Area Under the Receiver Operator Characteristic Curve (ROC) evaluations for individual models, symbolized by algorithm and colored by evaluation metric filter. Algorithms include Gradient Boosted Models (GBMs), Generalized Linear Models (GLMs), and RandomForests (RFs). Clustered points were jittered for better visibility.

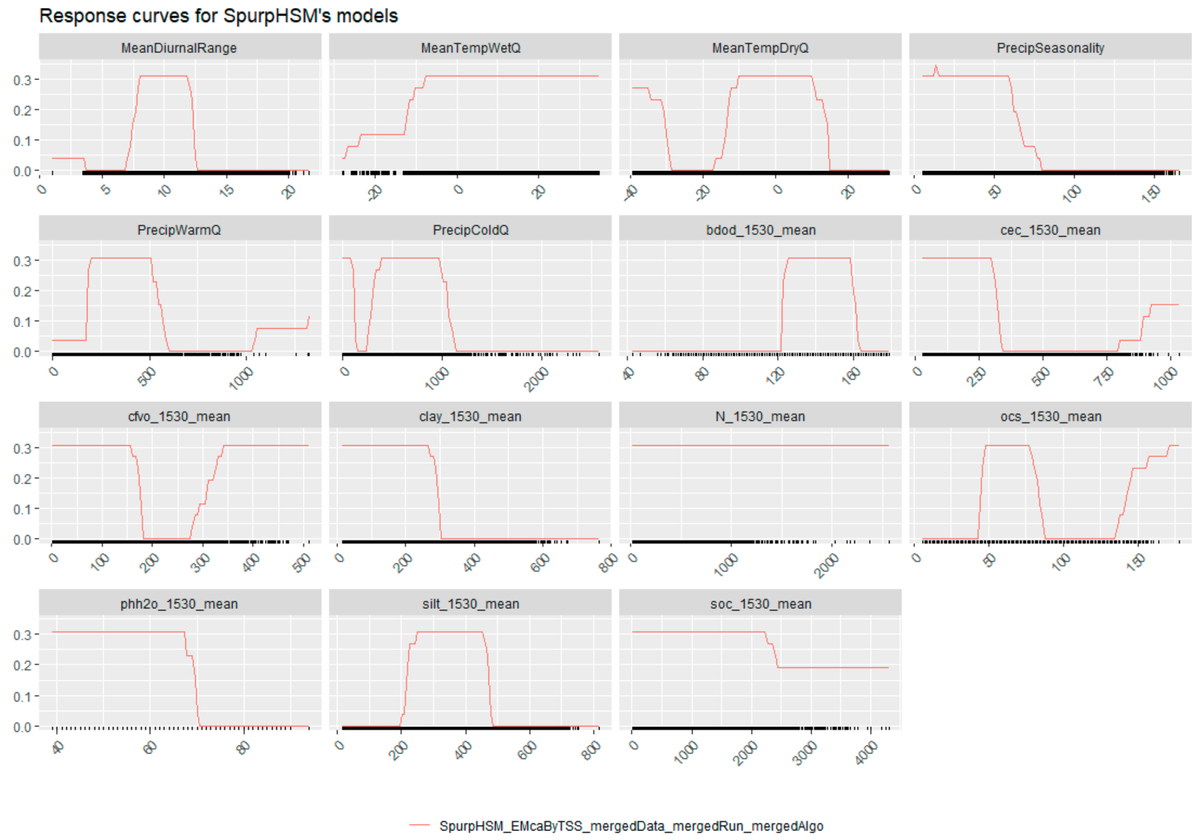

Figure S4 Response curves of *Sarracenia purpurea* habitat suitability to changes in environmental variables in the committee average ensemble model for environmental variables. Focal variables were held at their median values when implementing the evaluation strip method. Refer to Table S1 for variable descriptions and x-axis units.

SUPPLEMENTAL TABLES

Table S1 Environmental variable codes, their descriptions and units, and Variance Inflation Factor (VIF) analysis scores.

Variable Code	Variable Description	VIF Score
AnnMeanTemp	Annual Mean Temperature (°C)	>5
MeanDiurnalRange	Mean Diurnal Range (°C)	2.47
Isothermality	Isothermality (°C)	>5
TempSeasonality	Temperature Seasonality (°C)	>5
MaxTempWM	Maximum Temperature of the Warmest Month (°C)	>5
MinTempCM	Minimum Temperature of the Coldest Month (°C)	>5
TempAnnualRange	Temperature Annual Range (°C)	>5
MeanTempWetQ	Mean Temperature of the Wettest Quarter (°C)	2.89
MeanTempDryQ	Mean Temperature of the Driest Quarter (°C)	2.73
MeanTempWarmQ	Mean Temperature of the Warmest Quarter (°C)	>5
MeanTempColdQ	Mean Temperature of the Coldest Quarter (°C)	>5
AnnualPrecip	Annual Precipitation (mm)	>5
PrecipWetM	Precipitation of the Wettest Month (mm)	>5
PrecipDryM	Precipitation of the Driest Month (mm)	>5
PrecipSeasonality	Precipitation Seasonality (mm)	1.76
PrecipWetQ	Precipitation of the Wettest Quarter (mm)	>5
PrecipDryQ	Precipitation of the Driest Quarter (mm)	>5
PrecipWarmQ	Precipitation of the Warmest Quarter (mm)	2.65
PrecipColdQ	Precipitation of the Coldest Quarter (mm)	1.82
Bdod	Bulk Density of the Fine Earth Fraction (cg/cm^3)	3.42
Cec	Cation Exchange Capacity of the Soil (mmol(c)/kg)	2.11
Cfvo	Volumetric fraction of coarse fragments (cm^3/dm^3, volume %)	1.61
Clay	Proportion of Clay Particles (<0.0002 mm) in the Fine Earth Fraction (g/kg	1.53
N	Total Nitrogen (N) (cg/kg)	3.30
Ocd	Organic Carbon Density (hg/m^3)	>5
Ocs	Organic Carbon Stocks (t/ha	3.26
Phh2o	Soil pH (pH x 10)	2.89
Sand	Proportion of Sand Particles (>0.05/0.063 mm) in the Fine Earth Fraction (g/kg)	>5
Silt	Proportion of Silt Particles (≥ 0.002 mm and ≤0.05/0.063 mm) in the Fine Earth Fraction (g/kg)	1.51
Soc	Soil Organic Carbon Content in the Fine Earth Fraction (dg/kg)	3.12
